# A Systematic Overview of Type II and III Toxin-Antitoxin Systems with a Focus on Druggability

**DOI:** 10.3390/toxins10120515

**Published:** 2018-12-04

**Authors:** Sung-Min Kang, Do-Hee Kim, Chenglong Jin, Bong-Jin Lee

**Affiliations:** The Research Institute of Pharmaceutical Sciences, College of Pharmacy, Seoul National University, Gwanak-gu, Seoul 08826, Korea; men0528@snu.ac.kr (S.-M.K.); ozmagic15@snu.ac.kr (D.-H.K.); senglyong@snu.ac.kr (C.J.)

**Keywords:** toxin-antitoxin system, type II, type III, drug target, antimicrobial peptides, microbiota

## Abstract

Toxin-antitoxin (TA) systems are known to play various roles in physiological processes, such as gene regulation, growth arrest and survival, in bacteria exposed to environmental stress. Type II TA systems comprise natural complexes consisting of protein toxins and antitoxins. Each toxin and antitoxin participates in distinct regulatory mechanisms depending on the type of TA system. Recently, peptides designed by mimicking the interfaces between TA complexes showed its potential to activate the activity of toxin by competing its binding counterparts. Type II TA systems occur more often in pathogenic bacteria than in their nonpathogenic kin. Therefore, they can be possible drug targets, because of their high abundance in some pathogenic bacteria, such as *Mycobacterium tuberculosis*. In addition, recent bioinformatic analyses have shown that type III TA systems are highly abundant in the intestinal microbiota, and recent clinical studies have shown that the intestinal microbiota is linked to inflammatory diseases, obesity and even several types of cancer. We therefore focused on exploring the putative relationship between intestinal microbiota-related human diseases and type III TA systems. In this paper, we review and discuss the development of possible druggable materials based on the mechanism of type II and type III TA system.

## 1. Introduction

Toxin-antitoxin (TA) systems were originally discovered as plasmid maintenance systems possessed by almost all free-living bacteria in which only daughter cells harboring the TA operon can survive. Therefore, the TA genes are transferred vertically to daughter cells, a mechanism by which virulence and antibiotic resistance in pathogenic bacteria can be passed to new cells [1,2,3,4,5]. Since these discoveries, the importance of TA systems as a type of unit that performs a variety of alternative functions has been supported by increasing evidence. TA systems are involved in numerous physiological activities, including antibiotic resistance, postsegregational cell killing, cell dormancy, cell persistence, biofilm formation, cell division, DNA replication, translation, cell wall synthesis and the maintenance of membrane integrity [6,7,8,9,10,11]. Over the last decade, it has become possible to confirm these functional characteristics and exploit biochemical information to develop a new class of small molecule or small peptide antibiotics that target bacterial virulence [12,13].

TA systems consist of two components: A stable toxin and a labile antitoxin. In most cases, the toxins are proteins, while the antitoxins can be protein or RNA [3,14]. Recently, TA systems have been categorized into six types (I—VI) according to the nature of the antitoxin and the mechanism by which it regulates the toxin [15,16]. In type I and III TA systems, RNA antitoxins regulate the active toxin protein by inhibiting the translation of the toxin mRNA (type I) or by directly inhibiting toxin protein (type III). In type II and IV TA systems, antitoxins are proteins that directly bind and inhibit the toxin protein (type II) or indirectly counteract the toxin protein (type IV). The type V TA system antitoxin protein is an RNase that cleaves the mRNA encoding the toxin protein [17]. In type VI TA systems, the toxin protein is degraded by a specific protease in a complex with the antitoxin protein [18].

**Figure 1:**
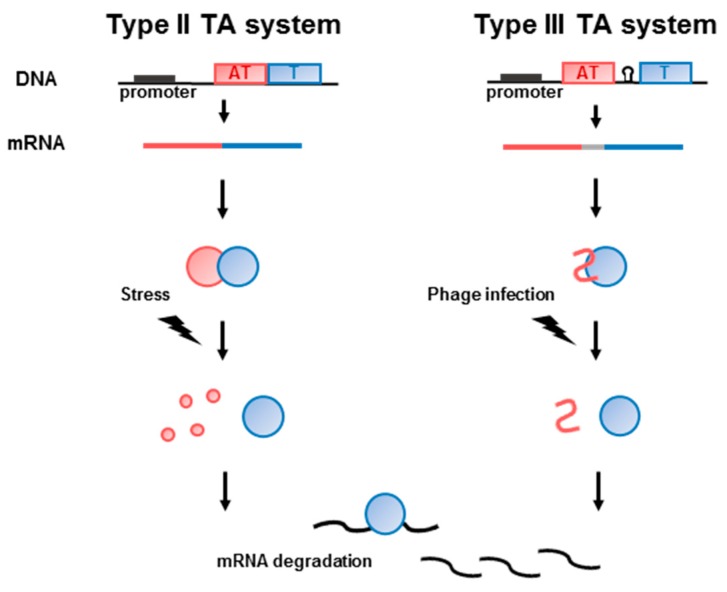
Schematic overview of type II and III Toxin-antitoxin (TA) system. The transcripts of TA system were expressed from the promoter of the operon. Toxin and antitoxin are defined as blue and red colors, respectively. The promoter is represented as a black box in the DNA. In type III system, antitoxin gene is separated with toxin gene by a Rho-independent terminator (represented as loop symbol) which regulate the toxin expression. Expressed toxin protein is neutralized by antitoxin protein (type II) or RNA (type III). When the external stimuli are applied, the toxin proteins are activated and degrade mRNA resulting in cell death.

Most toxin genes exist as operons with the cognate antitoxin genes, leading to transcriptional coupling. The antitoxin acts as a tight-binding inhibitor to block the toxicity of the toxin in type II and type III TA systems [9,19]. However, unfavorable circumstances, such as nutrient deficiency, antibiotic treatment, environmental stress, plasmid loss, bacteriophage infection, immune system attack, oxidative stress, or high temperature, can decrease the antitoxin concentration, leading to increased levels of free toxin and subsequent growth arrest and eventual cell death [19,20,21,22,23,24,25] (Figure 1).

Recently, controversy has arisen regarding the link between TA systems and persistence following exposure to antibiotics. Many TA systems have been implicated in persister formation. Toxins modulate the number of plasmids by inducing programmed cell death via postsegregational killing, and a lack of high toxin expression will eventually lead to persister cell formation or dormancy [26,27,28]. However, the results of recent studies have suggested that the evidence for postsegregational killing, persistence and phage inhibition is too weak to support the notion of a connection with TA systems. Persister formation has the potential to cause chronic infections. It causes antibiotic resistance or induces bacteria to adopt a quiescent state, which leads to persistence, in response to antibiotic treatment; therefore, reexamining the relevant functions of TA systems in light of the current controversy is of paramount importance [29,30].

**Figure 2:**
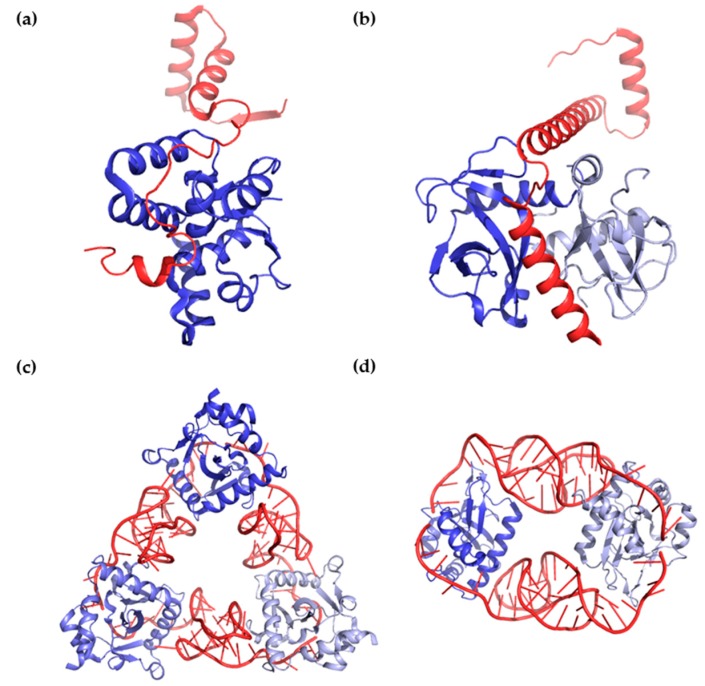
Binding and interaction modes of antitoxins and toxins.; (**a**,**b**) Structural views of binding between protein antitoxins and protein toxins. (**a**) The antitoxin VapB26 (red) and toxin VapC26 (blue) from *Mycobacterium tuberculosis* interact in a 1:1 ratio (PDB code 5X3T).; (**b**) The antitoxin MazE4 (red) and toxin MazF4 (blue and light blue) from *M. tuberculosis* interact in a 1:2 ratio (PDB code 5XE3); (**c**,**d**) Structural views of binding between RNA antitoxins and protein toxins; (**c**) The antitoxin ToxI (red) and toxin ToxN (blue colors) from *Pectobacterium atrosepticum* interact in a 1:1 ratio (PDB code 2XDD); (**d**) The antitoxin CptI (red) and toxin CptN (blue colors) from *Eubacterium rectal* interact in a 1:1 ratio (PDB code 4RMO).

In this paper, we review the biochemical, structural and functional data regarding type II and type III TA systems. In past decades, several structural and physiological studies have been performed to explore type II TA systems, while type III TA systems have only recently been studied. Protein toxins interact with protein (type II) or RNA (type III) antitoxins. Both types of antitoxins sterically block the active site of the toxin. However, in some cases, protein antitoxins can interact with more than one toxin to form 1:1 or 1:2 oligomers, whereas RNA antitoxins interact with toxins to form equimolar cyclic complexes [31,32,33,34] (Figure 2).

Recent studies have reported the rational design of peptides based on the binding interfaces of type II TA complexes. These peptides artificially activate toxins that were originally inactivated by their cognate antitoxin via competitive binding [31,35,36,37]. There are also other attractive possibilities, such as the antimicrobial agents involved in zeta-epsilon systems, which use synthetic peptides and small compounds. Liberated zeta toxin can block peptidoglycan synthesis followed by cell wall autolysis [38,39,40]. In addition, the human intestinal microbiota contains many organisms that possess type III TA systems, which are currently attracting attention because of their relevance in various human diseases, such as obesity, inflammatory and metabolic diseases and gastrointestinal and colon cancer, although type III systems do not necessarily cause these diseases [14,41,42,43,44,45]. Currently, 125 type III systems have been identified and categorized into three independent subtypes (*toxIN*, *cptIN* and *tenpIN*) that are distributed in many bacteria. Among these, 45 type III TA systems are present in the human microbiota [41,46]. The purpose of this review is to summarize the results of mechanistic and bioinformatic analyses of type II and type III TA systems with a focus on the potential druggability of these systems.

## 2. Type II TA Systems in Pathogenic Bacteria

Type II TA systems are abundant in almost all prokaryotes, especially pathogenic bacteria. Additionally, there is ample evidence of correlations between TA systems and bacterial pathogenicity [25,47,48,49,50,51,52]. Emerging evidence indicates that TA systems play fundamental roles in bacterial persistence and biofilm formation. A compound capable of corrupting dormant cells was shown to be able to kill persisters and eradicate a chronic biofilm infection [53]. Toxin expression has the potential to induce persister formation. Persistent cells are in a state of dormancy and are therefore able to survive under unfavorable conditions, including starvation, oxidative stress, and exposure to most antibiotics [5,54,55,56,57]. In exploiting bacterial toxins to induce cell killing, the induction of persistence should be avoided. Zeta toxins cause cell wall autolysis by substantially perturbing peptidoglycan synthesis [38,39,40], and bacterial mRNA endonuclease MazF toxin is suggested to be involved in programmed cell death. The programmed cell death induced by the toxins in TA systems is essential for therapeutic strategies [58,59], although controversy persists in the recent literature. There is the opinion that MazF only induces reversible inhibition of growth [29]. Although the claim of programmed cell death by MazF has been reported in recent literature [60], the programmed cell death by MazF is thought to be caused by artificial overproduction of MazF. Thus, in physiological levels for wild-type, MazF does not cause programmed cell death [61,62]. But other evidence shows that MazF toxin also causes tumor regression and regulates virulence factors. The anti-mazF peptide nucleic acids reduced virulence gene level, which demonstrated the association between TA systems and virulence factor. Also, solid tumors in mice were regressed upon induction of MazF [63,64]. In addition, TA systems influence environmental stress-induced biofilm formation. Bacterial biofilms are involved in numerous human chronic inflammatory and infectious diseases as well as in multidrug tolerance and resistance to the host immune system [24,52,54,65]. Furthermore, TA systems support the survival of pathogens within their hosts. In Salmonella, the majority of 14 type II TA modules are involved in the formation of persister cells and the induction of virulence factors [66].

Pathogenic bacteria that possess many TA systems include Staphylococcus aureus, Klebsiella pneumoniae, Pseudomonas aeruginosa, M. tuberculosis, Streptococcus pneumoniae, and Salmonella typhimurium. S. aureus, K. pneumoniae and P. aeruginosa are notorious for their antibiotic resistance, as emphasized by the Infectious Diseases Society of America (IDSA). Moreover, tuberculosis and pneumonia are severe infectious diseases associated with harmful invasive processes, and S. typhimurium is a major foodborne pathogen [67,68,69]. The current statistics regarding the type II TA systems in the above mentioned pathogens are shown in Table 1. There is one MazEF family toxin structure in S. aureus [70], and there are four in M. tuberculosis, including MazF3 [71], MazF6, MazF7 [72] and MazF9. MazEF4 possesses a complex structure that includes MazE4 and MazF4 [32]. Additionally, there are two RelBE family complexes in M. tuberculosis: RelBE2 and RelBE3. There are two complete VapBC family complex structures, VapBC2 and VapBC26 [31,73], and three complex structures in which the VapB antitoxin lacks the N-terminus, including VapBC5, VapBC15 and VapBC30 [36,74,75]. Only two VapC toxin structures, VapC20 and VapC21 [76,77], and only one VapB antitoxin structure, VapB45, have been reported. Only one HicBA complex structure has been reported in S. pneumoniae [37]. Regarding three RelBE loci of S. pneumoniae, The RelBE1 is inactive and has been considered as, probably, a defective system. The RelBE3 is, in fact, the YefM/YoeB pneumococcal TA. Then there is only one bona fides RelBE [78].

## 3. Application of Antimicrobial Peptides Based on the Type II TA Interface

Infectious diseases caused by antibiotic-resistant pathogenic bacteria are considered a major reason for human mortality worldwide. Importantly, TA systems are widely present in most important bacterial pathogens, but not in eukaryotic cells; they are therefore being evaluated as promising antibacterial targets [82,83]. Accumulating structural and functional data regarding TA systems identified in pathogenic bacteria have enabled the ‘artificial activation of toxins’ as a novel antibiotic strategy in which specific pathogens are targeted at the interface of a specific TA system. Usually, small molecule compounds or peptides are selected to increase toxin activity because they act as inhibitors of TA interactions. Peptides obtained from various sources have already been investigated for their ability to act as antibacterial agents and to replace existing antibiotics [84,85]. In most cases, toxins do not exhibit toxicity when they are part of a TA complex because the antitoxin completely blocks the active site of the toxin. However, when a peptide binds to a TA complex and interacts with its binding partners, the complex is disrupted by the peptide inhibitor, and the toxin is released from the complex. Because many of the toxins in type II TA systems are ribonucleases, the inhibitory potency of candidate peptides can be assessed by comparing the ribonuclease activity of the TA complex before and after the addition of the peptide inhibitors [86,87]. Alternatively, drug candidates that bind to the promoter region of a TA operon and thereby interfere with its transcription may represent another strategy. If a biomolecule with a high binding affinity for the TA operon binds to the promoter DNA, it will repress TA gene transcription. This type of nucleic acid inhibitor is constructed based on the DNA-binding region of an antitoxin and acts to hinder the supply of antitoxin. Because antitoxins neutralize the toxic effect of toxins in most type II TA systems, the host depends on the continuous cellular expression of the antitoxin. Although the toxin is thermodynamically stable, the antitoxin is unstable and is susceptible to cellular proteases. Consequently, the antitoxin has a lower half-life than the toxin. Therefore, repression of TA gene transcription and antitoxin supply results in free-toxin-induced cell lethality [3].

**Figure 3:**
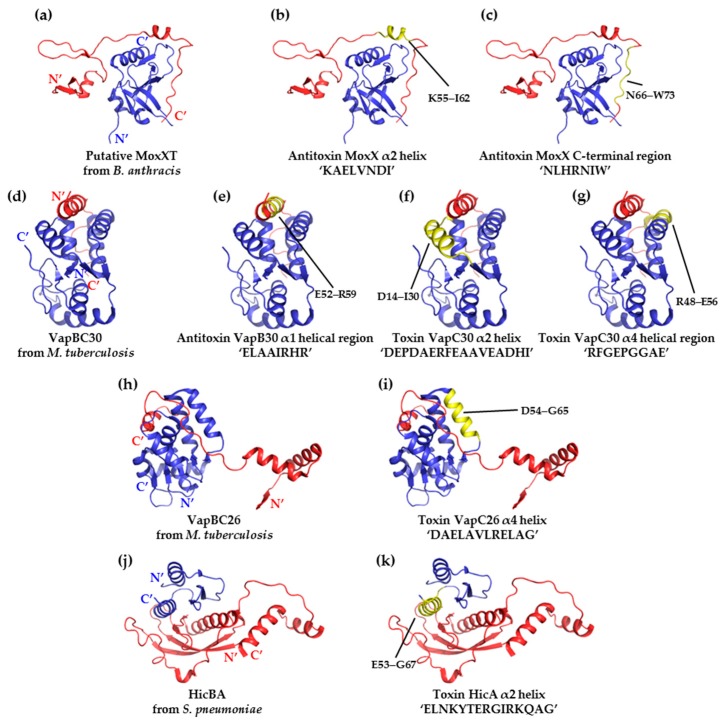
Ribbon representations of the structures of type II TA complexes used to design antimicrobial peptides, showing the target-binding site. Antitoxins (red), toxins (blue), and target-binding sites (yellow) are presented in different colors. The peptides and their sequences are also shown in the figure. (**a**) Putative MoxXT complex of *B. anthracis* (PDB code 1UB4 and 4HKE); target sites; (**b**) ‘KAELVNDI’ and (**c**) ‘NLHRNIW’; (**d**) VapBC30 complex of *M. tuberculosis* (PDB code 4XGQ); target sites; I ‘ELAAIRHR’; (**f**) ‘DEPDAERFEAAVEADHI’ and (**g**) ‘RFGEPGGAE’; (**h**) VapBC26 complex of *M. tuberculosis* (PDB code 5X3T); (**i**) target site ‘DAELAVLRELAG’; (**j**) HicBA complex of *S. pneumoniae* (PDB code 5YRZ); and (**k**) target site ‘ELNKYTERGIRKQAG’.

There is ample experimental evidence supporting the hypothesis that toxins can be artificially activated by peptides that mimic the toxin-binding region of an antitoxin. First, two peptides that mimic the C-terminal toxin-binding region of the antitoxin MoxX, which was obtained from *Bacillus anthracis*, exerted an approximately 20% inhibitory effect on the interaction between MoxX and MoxT. The inhibitory potency was calculated as the % inhibitory effect using the relative ribonuclease activity, which ranged from ‘toxin in complex’ (0) to ‘free toxin’ (100). In this type of assay, ribonuclease activity is measured using RNA probes containing fluorophores that emit fluorescence when cleaved by the ribonuclease toxin. If an adequate amount of peptidomimetic (corresponding to the concentration of the complexed protein) is used in the assay and enough time is allowed, the efficacy of the peptide can be estimated by measuring fluorescence. A ‘toxin in complex’ does not generate any fluorescence, because the toxicity of the toxin is completely inhibited by binding to the cognate antitoxin. In contrast, the addition of a peptidomimetic can lead to the formation of a ‘free toxin’ as a result of competitive binding, thereby generating fluorescence. First, the original peptides based on the MoxXT system were designed according to the structural homolog (PDB codes 1UB4) available for the *Escherichia coli* system [88]; however, the rationale for the design of these peptides was subsequently supplemented with biochemical data and data regarding the structure of the MoxT toxin (PDB code 4HKE) [35,89,90] (Figure 3a–c). Second, on the basis of the VapBC30 crystal structure (PDB code 4XGQ) from *M. tuberculosis*, three peptides (one antitoxin VapB30-mimicking peptide and two toxin VapC30-mimicking peptides) were designed. The inhibitory potency of the toxin-mimicking peptides was higher (73%) than that of the antitoxin-mimicking derivatives (43%) [36]. The peptides were designed to include key residues that interact with the interface between VapB30 and VapC30 (Figure 3a–g). Similarly, a toxin-mimicking peptide that targeted the VapBC26 complex of *M. tuberculosis* (PDB code 5X3T) exhibited an inhibitory potency of approximately 82% [31] (Figure 3h,i). Finally, in the *S. pneumoniae* HicBA system (PDB code 5YRZ), the toxin-mimicking peptide exerted a similar inhibitory potency of approximately 80% [37] (Figure 3j,k).The peptide that had the greatest TA complex-disrupting effect in the VapBC26 and HicBA systems is introduced in this review and in the main text of the original research papers. Interestingly, most successful peptide inhibitors mimic the α-helical region of the binding interface, indicating the importance of the structural nature of these peptides in the potential strength of their drug-like properties [91,92]. Indeed, the HicBA derivative peptide mimicking the longest helix among the listed peptides showed a much higher inhibition activity than other peptides. Although we cannot compare the binding ability of different TA systems, it is clear that peptide having a helical nature with an appropriate length is very important in peptide inhibitor potency. Additional information regarding the peptides mentioned in this paragraph is provided in Table 2.

## 4. Type III TA Systems in the Human Intestinal Microbiota

Type III TA systems have a substantial impact on the survival and physiological activities of the bacteria that harbor these systems, similar to the type II TA systems described above [14,41]. More than 90% of type III systems are conserved among three bacterial phyla: Firmicutes, Fusobacteria and Proteobacteria [41,46]. The functional unit of the type III TA system is characterized by RNA (antitoxin)-protein (toxin) interactions [9,93]. At the genetic level, the RNA antitoxin genes are located in the bicistronic operons upstream of the toxin genes and are transcribed along with the toxins by a single promoter [94,95]. Antitoxins of the type III TA system are composed of several tandem repeats of short nucleotide sequences and are cleaved by toxins, resulting in the formation of heteromeric complexes with the toxins [33,96].

Toxins of type III TA systems exhibit endoribonuclease activity and can cut their cognate antitoxin and other vital cellular mRNAs. These toxins form macromolecular complexes with the cognate RNA antitoxin in a pseudoknot conformation [33,41,46]. Type III TA systems participate in two major biological functions in bacteria. Their first function is abortive infection activity, which induces apoptosis in bacteria as a defensive mechanism against phages [94,97,98]. From an evolutionary perspective, bacteria reduce their population size by self-poisoning phage-infected cells to prevent phage propagation. When a bacteriophage invades a bacterium, type III toxins are activated and act as self-poisoning proteins, thus restricting the dissemination of phage progeny [99,100]. Second, plasmid stabilization is achieved by a plasmid addiction mechanism. Because of their labile nature, antitoxins must be continuously synthesized to prevent toxins from exerting their toxic effects. In other words, to survive under normal conditions, bacteria must be addicted to their own TA system [4,101,102]. Similarly, type III TA systems allow bacteria to adapt to this addiction mechanism and engage in essential activities, including persistence and biofilm formation [103,104].

The data previously deposited in a bioinformatic database indicates that there are 125 putative type III systems [41]. Type II TA systems are found in many pathogenic bacteria, and their relationships with pathogenicity have been intensively studied [5]. However, type III TA systems are strictly limited to particular pathogenic bacteria. Interestingly, type III TA systems are present in many bacteria that reside in the human intestinal microbiota. Therefore, type III TA systems may be associated with the physiological activity, survival, and essential cellular processes of bacteria in the intestinal microbiota [41,46]. The genera in the intestinal microbiota that harbor type III TA systems are *Marvinbryantia*, *Clostridium*, *Coprobacillus*, *Eubacterium*, *Fusobacterium*, *Lachnospiraceae*, *Lactobacillus*, *Phascolarctobacterium*, *Roseburia* and *Ruminococcus*. The 45 type III loci that have been identified in the intestinal microbiota are believed to account for more than one-third of all type III TA systems [14,41]. In general, the growth of microorganisms is regulated by TA systems, but globally, studies aimed at exploring the relationships between intestinal microorganisms and TA systems are at an early stage. The type III TA systems present in the human gastrointestinal microbiota, the lengths of the protein toxins and nucleotide antitoxins and the number of tandem repeats possessed by each are listed in Table 3. In type III TA system, toxins are protein, showing endoribonuclease and their cognate antitoxins are RNA, composed of several tandem repeats of short nucleotide sequences in the upstream of the toxin gene. In Table 3 for example, length ‘172 / 34 (2.9)’ means that protein toxin having 172 amino acids interacts with pseudoknot conformation of RNA antitoxin having 2.9 repeats of 36 nucleotides.

The significance of the association between the microbial environment and human diseases has been recently reported and has become a major research topic in related fields. The term ‘human intestinal microbiota’ refers to clusters of various microorganisms, such as bacteria, fungi, and protozoa, that are present in human intestines. The genes possessed by all members of the microbiota are called the ‘microbiome’, which is considered the ‘second genome’ of humans [105,106]. There are many microorganisms in the human intestine, and the intestinal microbiota is associated with the complex physiological activities of humans [107,108]. The intestinal microbiota affects human health and diseases by participating in digestion, the maintenance of intestinal homeostasis and the control of the metabolic balance via various mechanisms [109,110,111]. Consequently, the imbalance in the intestinal microbiota has been associated with inflammatory intestinal diseases, as well as obesity, diabetes, autoimmune and infectious diseases and even colorectal cancer. Therefore, the composition of an individual’s microbial taxa could be an important new diagnostic tool in a variety of diseases, and analyses of the relationships between humans and microbiota may provide breakthrough evidence to support the development of groundbreaking new therapeutic techniques [112,113,114,115,116].

Among the microorganisms that contain type III TA systems, *Marvinbryantia formatexigens* boosts the yield of succinate during acetogenesis. *M. formatexigens* ferments carbohydrates that cannot be digested by hosts and uses succinate, thereby assisting in the production of acetate, which is its main metabolic product [117,118]. Additionally, *Phascolarctobacterium* species use succinate as a substrate to produce propionate and synthesize ATP [119,120]. In addition, intestinal species in the genus *Clostridium*, including *Clostridium hiranonis*, use dehydroxylate bile acid to yield secondary bile acids, which are strong carcinogens associated with colorectal cancer [121,122,123]. Additionally, the proportions of *Coprobacillus* and *Coprococcus* species differed significantly between the fecal microbiota of subjects with irritable bowel syndrome and of healthy subjects [124]. Intriguingly, high-abundance therapy with *Eubacterium rectal* ameliorated inflammatory bowel disease, and *Eubacterium ventriosum* and *Ruminococcus torgues* were found to be enriched in patients with age-related macular degeneration [125,126,127]. *Fusobacterium* is involved in the migration of myeloid cells, participate in cancer induction and are widely distributed in tumors. *Fusobacterium* also induces inflammation and malignant tumor formation and causes inflammatory bowel diseases, such as appendicitis and colorectal cancer [128,129,130,131]. A recent study suggested that an abundance of *Fusobacterium* and *Clostridium* in the microbiota also increased the risk of gastric oncogenesis [132]. In contrast, butyrate-producing bacterial species in the genera *Lachnospiraceae*, *Roseburia* and *Ruminococcus* may protect against colorectal cancer and could be used as novel therapeutics to treat patients with Crohn’s disease [133,134,135,136,137]. Moreover, *Lactobacillus helveticus* has antitumor effects and enhances immune system activities [44,138,139,140]. *Lactobacillus* modulates pH and forms an adhesion layer on epithelial cells, making it difficult for pathogenic bacteria, such as *Staphylococcus*, to survive [141,142]. In obesity and diabetes, leptin levels were positively correlated with the enrichment of *Lachnospiraceae* and *Ruminococcus*. Additionally, *Coprococcus catus* and *E. ventriosum* were considerably more abundant in an obese group than in a nonobese group [143,144]. *Roseburia intestinalis* and *E. rectale* were present in lower abundance in a group with diabetes [145,146]. *Ruminococcus lactaris* was enriched in the gut in a group with rheumatoid arthritis [147]. Correlations between the composition of the intestinal microbiota and human diseases or functions are described in this section and listed in Table 3.

**Figure 4:**
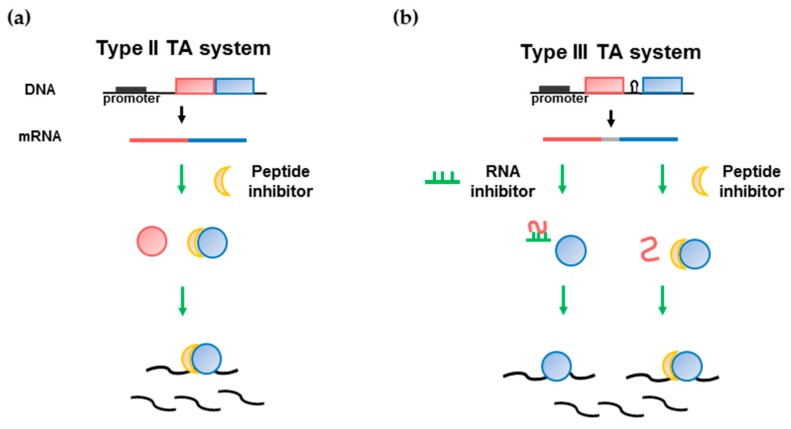
Drug development strategy targeting type II and III TA system. (**a**) Type II TA system. The peptide that binds to toxin inhibits the interaction with antitoxin protein.; (**b**) Type III TA system. The RNA oligomer that binds to the RNA antitoxin or peptide that binds to the toxin prohibiting the interaction with RNA antitoxin inhibits the toxin protein binding to RNA antitoxin. The active toxin proteins of both TA system bind to the mRNA and cleavage it resulting in cell death. Toxin and antitoxin are defined as blue and red colors, respectively.

Although several of the microbiota species that possess type III TA systems are known to be related to diseases, as shown above, no study has explored drug discovery with the aim of targeting a type III TA system. Therefore, drugs based on type III TA systems could be developed as novel drug candidates associated with cancer or other adult diseases. In type II TA system, peptides are mainly considered as inhibitors for the discovery of therapeutic peptides (Figure 4a). A strategy to develop drugs that target type III TA systems could be established by exploring the ability to activate protein toxins by using RNAs or peptides to disrupt the interaction between toxin proteins and antitoxin RNAs (Figure 4b). First, an RNA inhibitor can be developed for type III TA system. There was an example for an RNA inhibitor studied for the *hok*/*sok* type I TA system by Faridani et al. [148]. An anti-Sok peptide nucleic acid (PNA) oligomer inhibited *hok* mRNA:Sok-RNA interactions, resulting in cell killing through the synthesis of the Hok protein. Based on the available structural information regarding type III TA complexes, a short nucleic acid oligomer can be constructed to bind to the protein toxin and block the region at which the antitoxin RNA binds to the toxin protein. Second, peptide inhibitors can be designed as binding inhibitors based on the binding interface of type II TA systems. Peptides that mimic the RNA binding site of a toxin could hinder protein-RNA interactions. Inhibitors derived using the above strategy could therefore result in toxin activation. The inhibitory potency of an inhibitor can be evaluated using the endoRNase activity of type III toxins, similar to assays performed for type II toxins. In some pathogenic microbiota members, if the type III toxin is activated by an inhibitor, it can act as an effective drug with a new mechanism. Inhibitors targeting cancer-causing microbiota members, such as *Clostridium* and *Fusobacterium* species may be used as drugs or drug conjugates to prevent cancer. Additionally, as described above, since many diseases are associated with imbalance in the intestinal microbial system, we may find useful clinical evidence for the pathogenesis of microbial dysbiosis through a cytotoxicity experiment that targets a specific species in the microbiota. Further studies of inhibitors of type III TA systems should be conducted to develop effective novel drug candidates [149,150].

## 5. Closing Remarks

Recent investigations of TA systems have highlighted the potential of TA systems as new druggable targets. The accumulated structural and functional data related to type II TA systems have provided useful insights that support the development of new antibiotics. Antimicrobial peptides with the ability to hyperactivate toxin activity under normal conditions in order to eradicate bacterial cells could be potential anti-infection agents. Because multidrug resistance makes infectious diseases very difficult to treat, the discovery of antibiotics based on TA systems that can disrupt TA complexes seems to be a promising pipeline for the development of antibacterial agents [82,83,151,152].

Currently, the increasing number of solved structures of type II and III TA systems supports the design of molecules that disrupt the TA interface. We have introduced several rational explanations of how to induce the artificial activation of toxins based on the currently available structural and biochemical information regarding type II and III TA systems. The peptides described in this study target the binding interface of the TA complex, especially the α-helical region. These peptides mimic each α-helical region in the binding interface and compete with their binding counterparts to release the free toxin from the TA complex. For these peptides to become drug candidates, limitations of peptide therapy should be considered. For examples, low oral bioavailability, short half-life, protease susceptibility, immunogenic effects, toxicity and high cost [153]. To improve these shortcomings, several methods have been tried. The modification of antimicrobial peptide with a conjugate, such as polymer, antibiotics or fatty acids can be applied to increase the antimicrobial activity of peptides [154]. Attaching antibacterial cell-penetrating peptides to peptide inhibitors of TA interactions may improve the delivery and permeability of peptide drug candidates [155]. Nanoparticles as potentially useful drug delivery systems that do not reduce the therapeutic efficacy of drugs also can be used. For example, antimicrobial peptides tethered to gold nanoparticles exhibited a maintained conformational flexibility and reduced protease susceptibility [156].

In addition, Tat protein-derived peptides may be another alternative because Tat peptides can efficiently ferry drug candidates into target tissue cells via chemical crosslinking. Tat-mediated delivery may allow the uptake of macromolecules into tissues that were previously thought to be impermeable [157,158].

To ensure toxin activation, antimicrobial peptides should have a strong binding affinity to the TA interface and be compatible with the toxin or antitoxin. Hence, surface modulation, such as stapling the peptides using hydrocarbon crosslinking or substituting the main chain hydrogens to constrain the peptides in an α-helical conformation, could increase the stability and activity of the peptides and increase their affinity for the target TA complex [159,160,161]. TA systems are emerging as targets of new antimicrobial compounds, and drugs based on TA systems are likely to be highly specific because mammalian hosts lack TA modules. For development as highly effective future therapeutic alternatives, drug candidates based on TA systems should be systemically validated and carefully monitored, because these drugs may have deleterious effects on normal commensal organisms or microbiota that may perform beneficial functions in the human body [3,162,163]. A TA system that is present in a target pathogenic bacterial species, but not in innocuous bacteria should be selected when selecting drug targets. If there are similar or identical systems in both the target bacteria and innocuous bacteria, drug candidates should be carefully designed based on structural information to avoid causing harm to innocuous bacteria. Additionally, because TA systems are abundant in many different bacteria, it is necessary to develop lead compounds that can simultaneously target, crossregulate and activate multiple TA systems and thereby act as broad-spectrum antibiotics [52,87].

Regarding type III TA systems, it is clear that further characterization of the molecular mechanisms involved in type III TA systems will help us understand the functions of the human intestinal microbiota. The intestinal microbiota supplies nutrients that cannot be produced by host enzymes and are closely associated with the metabolism and immune responses of the host [164,165,166]. The diseases associated with the microbiota are regulated by the equilibrium of the microbiota and imbalances therein. The microbiota maintains homeostasis in healthy hosts. However, when inflammation is induced in the body, due to pathogenic infection, nutritional imbalance, or severe stress, host tissue cells are destroyed, resulting in dysbiosis of the microbiota [115,167]. Microbiota that are modified in this way can be recovered by appropriate treatment, but in some cases, the modification is irreversible and can induce chronic disease. To date, the intestinal microbiota has been linked to the development of obesity and metabolic syndromes, including diabetes, inflammatory bowel disease, autoimmune disease and cancer [168,169,170]. However, with regard to the mechanisms underlying the onset of these diseases and symptoms, we are still in the preliminary stages of understanding whether alterations in the microbiota are direct causes or consequences of the diseases and identifying the intestinal microbes responsible for them. For microbiota with known correlations with disease states, a drug development strategy similar to that used in type II TA systems can also be applied. Because type III antitoxins are RNAs, it is possible to use either protein-based or RNA-based inhibitors of the TA complex. For example, *Fusobacterium* participates in cancer induction, and antimicrobial peptides or short nucleic acid oligomers based on its type III TA interface could be designed to inactivate target bacteria. Therefore, identifying the precise role of the microbiota and determining whether a specific microbe is harmful or beneficial will be indispensable to the study of various diseases. There may be as-yet-unidentified disease-related microbes that possess type III TA systems. The number of disease-related type III TA systems that have been discovered is expected to increase, and the details of their functions and molecular mechanisms will need to be revealed.

TA systems present in bacteria have biological functions that affect not only postsegregational killing and abortive infection, but also bacterial persistence [16]. Therefore, with regard to the druggability of compounds that are developed to activate the toxins in TA systems, we should consider bacterial persistence, which represents tolerance to antibiotics and exposure to other environmental stress conditions. One possible drawback is that fluctuations in the level of toxin activation could induce the formation of persister or dormant cells, leading to chronic infection [163,171].

Overall, TA systems have both industrial and academic value. We can imagine a scenario in which novel drugs could be based on type II TA systems. Studies that explore the causal relationships between the evolution of type III TA systems in the intestinal microbiota and human diseases are crucial to future research in the field of microbiology. In conclusion, the role of TA systems in the physiology of both pathogenic bacteria and the intestinal microbiota warrants further consideration.

## Figures and Tables

**Table 1:** Overview of the type II TA systems in pathogenic bacteria described in this review.

Pathogenic Bacteria	TA Pair (Antitoxin/Toxin)	Reported Structure	PDB Code	Ref.
*Staphylococcus aureus*	MazE/MazF	Toxin MazF	4MZM	[70]
	RelB/RelE (2 distinct loci)			
*Klebsiella pneumoniae*	HipB/HipA (2 distinct loci)			
	MazE/MazF			
	Phd/Doc			
	RelB/RelE (3 distinct loci)			
	VapB/VapC			
*Pseudomonas aeruginosa*	RelB/RelE			
	VapB/VapC			
*Mycobacterium tuberculosis*	HigA/HigB (2 distinct loci)			
	MazE/MazF (9 distinct loci)	Toxin MazF3	5CCA	[71]
		Complex MazEF4	5XE3	[32]
		Toxin MazF6	5HKC	
		Toxin MazF7	5WYG	[72]
		Toxin MazF9	5HJZ	
	ParD/ParE (2 distinct loci)			
	RelB/RelE (3 distinct loci)	Complex RelBE2	3G5O	
		Complex RelBE3	3OEI	
	VapB/VapC (51 distinct loci)	Complex VapBC2	3H87	[73]
		Complex VapBC5	3DBO	[74]
		Complex VapBC15	4CHG	[75]
		Toxin VapC20	5WZF	[76]
		Toxin VapC21	5SV2	[77]
		Complex VapBC26	5X3T	[31]
		Complex VapBC30	4XGQ	[36]
		Antitoxin VapB45	5AF3	
*Streptococcus pneumoniae*	HicB/HicA	Complex HicBA	5YRZ	[37]
	HigA/HigB			
	RelB/RelE (3 related loci)			[78]
	Phd/Doc			
*Salmonella typhimurium*	HigA/HigB			
	RelB/RelE (9 distinct loci)			
	Phd/Doc			
	VapB/VapC			

* This table contains information obtained from the TADB2, UniProt and Protein Data Bank databases [79,80,81].

**Table 2:** Overview of peptide inhibitors explored as drug candidates and described in this review.

Target System (PDB Code)	Region Being Mimicked (Residue Range)	Peptide Sequence	% Inhibition
MoxXT (MazEF) from *B. anthracis* (using structural homolog 1UB4)	Putative α2 helix of the toxin MazF (55–62)	KAELVNDI	22
	Putative C-terminal toxin-binding region of the antitoxin MazE (66–73)	NLHRNIW	20
VapBC30 from *M. tuberculosis* (4XGQ)	α1 helical region of the antitoxin VapB30 (52–59)	ELAAIRHR	43
	α2 helix of the toxin VapC30 (14–30)	DEPDAERFEAAVEADHI	53
	α4 helical region of the toxin VapC30 (48-56)	RFGEPGGRE	73
VapBC26 from *M. tuberculosis* (5X3T)	α4 helix of the toxin VapC26 (54–65)	DAELAVLRELAG	82
HicBA from *S. pneumoniae* (5YRZ)	α2 helix of the toxinHicA (53-67)	ELNKYTERGIRKQAG	80

* 4HKE was used to refine the peptidomimetics based on MoxXT.

**Table 3:** Type III TA systems in members of the human gastrointestinal microbiota.

Strain	Family	Length T/A (Repeat)	Related Functions or Diseases
*Marvinbryantia formatexigens* DSM 14469	*toxIN*	172/34 (2.9)	Acetogenesis [117,118]
	*toxIN*	182/38 (2.1)	
	*cptIN*	161/47 (2)	
	*cptIN*	66/45 (2)	
*Clostridium hiranonis* DSM 13275	*cptIN*	157/45 (2.2)	Colorectal cancer Gastric cancer [121,122,123,132]
	*tenpIN*	158/55 (2.1)	
*Clostridium nexile* DSM 1787	*toxIN*	129/38 (2.2)	
*Clostridium* sp. HGF2	*toxIN*	139/46 (2.1)	
	*cptIN*	161/47 (2.2)	
*Coprobacillus* sp. 29_1	*toxIN*	163/38 (2.2)	Irritable bowel syndrome [124]
*Coprococcus catus* GD/7	*cptIN*	160/46 (2.2)	Irritable bowel syndrome Obesity [124,143,144]
*Coprococcus* sp. ART55/1	*toxIN*	181/37 (3.4)	
*Eubacterium rectale* ATCC 33656	*cptIN*	162/45 (2.1)	Inflammatory bowel disease Diabetes Macular degeneration Obesity [125,126,127,145,146]
	*cptIN*	158/46 (2.2)	
*Eubacterium rectale* DSM 17629	*toxIN*	201/38 (2.1)	
	*cptIN*	162/45 (2.1)	
*Eubacterium rectale* M104/1	*toxIN*	201/38 (2.1)	
*Eubacterium ventriosum* ATCC 27560	*cptIN*	162/46 (2.2)	
*Fusobacterium* sp. 2_1_31	*cptIN*	159/40 (2.9)	Inflammatory bowel disease Colorectal cancer Gastric cancer [128,129,130,131,132]
*Fusobacterium* sp. 3_1_33	*cptIN*	158/41 (3)	
	*tenpIN*	140/41 (3)	
*Fusobacterium* sp. 3_1_36A2	*tenpIN*	144/53 (2.1)	
*Fusobacterium* sp. 3_1_5R	*toxIN*	174/39 (2)	
	*toxIN*	178/38 (3.3)	
	*toxIN*	189/35 (3.2)	
*Fusobacterium* sp. 4_1_13	*toxIN*	179/39 (2)	
	*tenpIN*	144/53 (2.1)	
*Fusobacterium* sp. 7_1	*cptIN*	156/40 (3.1)	
*Fusobacterium* sp. D11	*cptIN*	158/40 (3.1)	
*Fusobacterium* sp. D12	*toxIN*	173/39 (2)	
*Fusobacterium ulcerans* ATCC 49185	*toxIN*	166/35 (3.2)	
*Lachnospiraceae bacterium* 2_1_46FAA	*toxIN*	163/38 (3)	Colorectal cancer Crohn’s disease Obesity [133,134,135,136,137,143,144]
	*toxIN*	163/38 (3.2)	
*Lachnospiraceae bacterium* 4_1_37FAA	*toxIN*	163/38 (3.2)	
*Lachnospiraceae bacterium* 5_1_63FAA	*cptIN*	162/46 (2.2)	
*Lachnospiraceae bacterium* 8_1_57FAA	*toxIN*	163/38 (3.2)	
*Lachnospiraceae bacterium* 9_1_43BFAA	*cptIN*	54/45 (2.2)	
*Lactobacillus helveticus* DSM 20075	*toxIN*	124/37 (1.9)	Immune enhancement [44,138,139,140] Antitumor
*Phascolarctobacterium* sp. YIT 12067	*cptIN*	162/46 (2.1)	ATP synthesis [119,120]
*Roseburia intestinalis* M50/1	*toxIN*	146/39 (3.2)	Colorectal cancer Crohn’s disease [133,134,135,136,137,145,146] Diabetes
*Roseburia intestinalis* XB6B4	*toxIN*	166/39 (3.2)	
*Ruminococcus lactaris* ATCC 29176	*cptIN*	162/46 (2.2)	Rheumatoid arthritis, Colorectal cancer, Crohn’s disease, obesity Macular degeneration [125,126,127,133,134,135,136,137,147]
*Ruminococcus* sp. 5_1_39B_FAA	*toxIN*	178/36 (2.1)	
*Ruminococcustorques* ATCC 27756	*toxIN*	163/38 (3.2)	
*Ruminococcus torques* L2-14	*cptIN*	162/46 (2.2)	

* The gastrointestinal microbiota contains 23 *toxIN*, 18 *cptIN* and 4 *tenpIN* loci among the 45 total type III TA systems. ‘Length’ refers to the number of amino acids of toxin and the number of nucleotides of antitoxin.
